# Sympathetic Hyperactivity, Increased Tyrosine Hydroxylase and Exaggerated Corpus Cavernosum Relaxations Associated with Oxidative Stress Plays a Major Role in the Penis Dysfunction in Townes Sickle Cell Mouse

**DOI:** 10.1371/journal.pone.0166291

**Published:** 2016-12-09

**Authors:** Fábio H. Silva, Mário A. Claudino, Fabiano B. Calmasini, Eduardo C. Alexandre, Carla Franco-Penteado, Arthur L. Burnett, Edson Antunes, Fernando F. Costa

**Affiliations:** 1 Hematology and Hemotherapy Center, University of Campinas, Faculty of Medical Sciences, University of Campinas, Campinas, SP, Brazil; 2 Laboratory of Multidisciplinary Research, São Francisco University Medical School, Bragança Paulista, SP, Brazil; 3 Department of Pharmacology, Faculty of Medical Sciences, University of Campinas, Campinas, SP, Brazil; 4 The James Buchanan Brady Urological Institute and Department of Urology, The Johns Hopkins School of Medicine, Baltimore, MD; Boston University, UNITED STATES

## Abstract

**Background:**

Sickle cell disease patients display priapism that may progress to erectile dysfunction. However, little is known about the pathophysiological alterations of corpus cavernosum in sickle cell disease.

**Objective:**

Thus, this study aimed to evaluate the functional and molecular alterations of sympathetic machinery and nitric oxide—cyclic guanosine monophosphate signaling pathway in Townes transgenic sickle cell disease mice.

**Methods:**

Concentration–response curves to contractile (phenylephrine) and relaxant agents (acetylcholine and sodium nitroprusside) were obtained in corpus cavernosum strips from sickle and C57BL/6 (control) mice. Neurogenic contractions and nitrergic relaxations were obtained using electrical-field stimulation. Measurements of endothelial nitric oxide synthase (eNOS), neuronal nitric oxide synthase (nNOS), phosphodiesterase-5 (PDE5) and α_1A_-, α_1B_- and α_1D_-adrenoceptor mRNA expressions and reactive-oxygen species were performed. Tyrosine hydroxylase phosphorylated at Ser-31 and total tyrosine hydroxylase protein expressions in cavernosal tissues were also measured.

**Results:**

The neurogenic contractions were higher in the sickle cell disease group, in association with elevated tyrosine hydroxylase phosphorylated at Ser-31 and total tyrosine hydroxylase protein expression, as well as increased tyrosine hydroxylase mRNA expression. Likewise, phenylephrine-induced contractions were greater in the sickle mice, whereas α_1A_-, α_1B_- and α_1D_-adrenoceptor mRNA expression remained unchanged. Cavernosal relaxations to acetylcholine, sodium nitroprusside and EFS were higher in sickle mice, accompanied by decreased eNOS and nNOS, along with lower PDE5 mRNA expression. An increase of about 40% in reactive-oxygen species generation in corpus cavernosum from sickle mice was also detected.

**Conclusion:**

Our study shows that decreased nitric oxide bioavailability in erectile tissue due to increased oxidative stress leads to both sympathetic hyperactivity and dysregulation of nitric oxide signaling in corpus cavernosum from Townes sickle mice.

## Introduction

Sickle cell disease (SCD), an inherited disorder of hemoglobin production, is a disorder of polymerization of hemoglobin S (HbS). HbS is caused by a mutation in the β-globin gene. Polymerization HbS within erythrocytes causes stiffness of red blood cells, resulting in hemolysis, vaso-occlusive crisis, stroke, pulmonary hypertension, osteonecrosis and leg ulcers [1]. Transgenic sickle cell mice have been employed to better understand the complex pathophysiology of SCD. Berkeley SCD mice exclusively express human sickle hemoglobin, and exhibit the major features found in human SCD such as vaso-occlusion and organ damage [2]. The homozygous Townes transgenic sickle cell mouse is another model for SCD that was developed by the replacement of the mouse α-globin genes by human α-globin genes, while the mouse β-globin genes are replaced by human Aγ and β^S^ (sickle) globin genes [3]. Homozygous Townes SCD mice develop disease symptoms such as severe anemia due to erythrocyte sickling, splenic infarcts, renal damage, liver damage, vaso-occlusion and urine concentration defects [3]. Moreover, priapism is highly prevalent in about 42% of male SCD patients [4]. Priapism is defined as a penile erection that persists beyond, or is unrelated, to sexual interest or stimulation that may progress to erectile dysfunction [5].

Penile vessels and cavernosal smooth muscle are supplied by a large number of sympathetic nerve terminations [6,7]. In the penile flaccid state, erectile tissue and penile vessels are contracted mainly via a tonic activity of neurotransmitter noradrenaline, released from the sympathetic nerves [8]. Tyrosine hydroxylase is the first rate-limiting enzyme in catecholamine biosynthesis and catalyzes the hydroxylation of tyrosine to L-dihydroxyphenylalanine (L-DOPA). Dihydroxyphenylalanine decarboxylase catalyzes the conversion of L-DOPA to dopamine, which in turn is converted to noradrenaline by dopamine β-hydroxylase [9]. Activation of α_1_-adrenoceptors by noradrenaline elicits phosphatidylinositol 4,5 bisphosphate hydrolysis and hence generation of the second messenger inositol 1,4,5-trisphosphate, which activates the inositol 1,4,5-trisphosphate receptor to release Ca^2+^ from sarcoplasmic reticulum. Intracellular Ca^2+^ binds to calmodulin, and this complex activates myosin light chain kinase, with subsequent phosphorylation of myosin light chain, resulting in smooth muscle contraction and a flaccid penis [10].

Nitric oxide (NO) is well established as a mediator of penile erection, and eNOS and nNOS isoforms serve as the source to produce NO in corpus cavernosum [11]. NO, released from nitrergic nerves and endothelial cells, activates the soluble guanylate cyclase enzyme in cavernosal smooth muscle, resulting in the accumulation of intracellular cyclic guanosine monophosphate (cGMP), which leads to relaxation of corpus cavernosum smooth muscle and penile erection [8]. Intracellular cGMP is rapidly inactivated to 5’GMP by phosphodiesterase-5 (PDE5), thus ceasing the erectile response [12]. In addition, evidence indicates that an excess of reactive-oxygen species (ROS) such as superoxide anion (O_2_^-^) contributes to impaired endothelium-dependent and nitrergic cavernosal relaxations by decreasing NO bioavailability, leading to erectile dysfunction [13–15]. As mentioned above, activation of the sympathetic nervous system and hence noradrenaline release is crucial to determine cavernosal smooth muscle contraction and penile detumescence, but no study has evaluated sympathetic neurotransmission in the corpus cavernosum of SCD mice. Therefore, we evaluated the sympathetic vasoconstriction and α_1_-adrenoceptor-mediated contractile responses, as well as the mRNA expressions for tyrosine hydroxylase and α_1_-adrenoceptors (α_1A_-, α_1B_- and α_1D_ subtypes) in the corpus cavernosum of Townes SCD mice. Moreover, as SCD is associated with elevated ROS production [16], we hypothesized that increased ROS levels in the corpus cavernosum of Townes SCD mice contribute to alterations in the NO-cGMP signaling pathway. Hence, functional assays focusing on the NO-mediated corpus cavernosum relaxations and mRNA expressions for endothelial nitric oxide synthase (eNOS), neuronal nitric oxide synthase (nNOS) and phosphodiesterase type 5 (PDE5) in the erectile tissue from Townes SCD mice were evaluated.

## Material and Methods

### Ethics Statement

All experimental procedures in this study were carried out in accordance with the general ethical guidelines for animal use established by the Brazilian Society of Laboratory Animal Science (SBCAL) and EC Directive 86/609/EEC for Animal Experiments and were approved by an institutional Committee for Ethics in Animal Experimentation of the University of Campinas (IACUC/CEEA-UNICAMP, Permit number 3909–1). The animals were euthanized with an overdose of sodium pentobarbital (1 mg/g) and all efforts were made to minimize animal suffering.

### Animals

All animal procedures and the experimental protocols were carried out according to the Ethical Principles in Animal Research adopted by the Brazilian College for Animal Experimentation and followed the Guide for the Care and Use of Laboratory Animals. C57BL/6 mice (wild type) and Townes transgenic sickle cell mice aged 3 to 4 months-old were used for these studies. The mice were obtained from Jackson Laboratories (Bar Harbor, ME) and were generated and characterized at the Multidisciplinary Center for the Investigation of Biological Science in Laboratory Animals of University of Campinas. The animals were housed three per cage on a 12 h light–dark cycle. The average weights of dry cavernosal strips from control and SCD mice were 9.2 ± 0.4 and 8.8 ± 0.4 mg, respectively.

### Functional studies in cavernosal strips and concentration-response curves

Mice were anesthetized with isoflurane and exsanguinated. Strips of mouse corpus cavernosum were mounted in a 10-ml organ system containing Krebs solution at 37°C, continuously bubbled with a mixture of 95% O_2_ and 5% CO_2_ (pH 7.4), and vertically suspended between two metal hooks. One hook was connected to a force transducer and the other acted as a fixed attachment point. Tissues were allowed to equilibrate for 60 min under a resting tension of 2.5 mN. Isometric force was recorded using a PowerLab 400^TM^ data acquisition system (Software LabChart, version 7.0, AD Instrument, MA, USA). Cumulative concentration–response curves to the α1-adrenoceptor agonist phenylephrine (10^−8^ to 3 × 10^−4^ M) were obtained in cavernosal strips. In separate experiments, cumulative concentration-response curves were constructed for both the muscarinic agonist acetylcholine (ACh; 10^−9^ to 10^−5^ M) and the NO-donor compound sodium nitroprusside (SNP; 10^−8^ to 10^−5^ M) in cavernosal strips pre-contracted with phenylephrine (3 × 10^−6^ to 10^−5^ M). Nonlinear regression analysis to determine the pEC_50_ was carried out using GraphPad Prism (GraphPad Software, San Diego, CA, USA).

### Electrical-field stimulation (EFS)

EFS was applied on cavernosal strips placed between two platinum ring electrodes connected to a Grass S88 stimulator (Astro-Med Industrial Park, RI, USA). EFS was conducted at 50 V, 1 ms pulse width and trains of stimuli lasting 10 sec at varying frequencies. In order to study the nitrergic cavernosal relaxations, tissues were pretreated with guanethidine (3 × 10^−5^ M; to deplete the catecholamine stores of adrenergic fibers) and atropine (10^−6^ M; to produce muscarinic antagonism) for 30 minutes prior to pre-contraction with phenylephrine [17]. When a stable contraction level was attained, a series of EFS-induced relaxations were constructed (1–32 Hz). In another set of experiments, to evaluate adrenergic nerve-mediated responses, the strips were incubated with N^ω^-nitro-L-arginine methyl ester, nonspecific NOS inhibitor; 10^−4^ M) plus atropine (10^−6^ M), before EFS was performed [18].

### ROS measurement

The oxidative fluorescent dye hydroethidine was used to evaluate *in situ* ROS generation [17]. Mice corpora cavernosa were embedded in a freezing medium and transverse sections (12 μm) of frozen tissue were obtained on a cryostat, collected on glass slides and equilibrated for 10 min in Hanks solution (pH 7.4) at 37°C. Fresh Hanks solution containing dihydroethidium (DHE; 2 × 10^−6^ M) was topically applied to each tissue section and the slices were incubated in a light-protected humidified chamber at 37°C for 30 min. Images were obtained with an Eclipse 80i microscope (Nikon, Japan) equipped for epifluorescence (excitation at 488 nm; emission at 610 nm) and camera (DS-U3, Kilon, Japan). Fluorescence was detected with a 585 nm long-pass filter. The number of nuclei labeled with ethidium bromide (EB-positive nuclei) along the cavernosal tissue was automatically counted using Image J Software (National Institute of Health, Bethesda-MD, USA) and expressed as labeled nuclei/mm^2^.

### Real time reverse transcription polymerase chain reaction (RT-PCR)

Total RNA was extracted with Trizol Reagent (Invitrogen Corp., Carlsbad, Ca, USA) from mouse corpus cavernosum samples. Three microgram RNA samples were incubated with 1U DNaseI (Invitrogen, Rockville, MD, USA) for 15 min at room temperature (RT) and ethylenediaminetetraacetic acid was added to a final concentration of 2 mM to stop the reaction. The DNaseI enzyme was subsequently inactivated by incubation at 65°C for 5 min. DNaseI-treated RNA samples were then reverse transcribed with Superscript III and RNaseOut (Invitrogen Corp., Carlsbad, Ca, USA) for 50 min at 50°C, and 15 min at 70°C. cDNA samples were quantified using a Nanodrop spectrophotometer (ND-1000; Nanodrop Technologies Inc., Wilmington, DE, USA). Primers were designed using the PrimerExpress^TM^ program (Applied Biosystems, Foster City, CA, USA) (Table 1). The ideal concentration of use was determined for each pair of primer and the amplification efficiency was calculated according to the equation *E*^*(-1/slope)*^, to confirm the accuracy and reproducibility of the reactions. Amplification specificity was verified by running a dissociation protocol. qRT-PCRs were performed in duplicate, using 6 μl SYBR Green Master Mix (Applied Biosystems), 10 ng cDNA and ideal quantities of each primer in a final volume of 12 μl. Samples were run in MicroAmp Optical 96-well plates (Applied Biosystems) in a 7500 Fast Real Time PCR System (Applied Biosystems). Gene expression was quantified using the Gnorm program. Results are expressed as mRNA levels of each gene studied, normalized according to β-actin and GAPDH expressions [19].

**Table 1:** Sequences and ideal concentrations for the primers used in qRT-PCR.

Gene	Primer sequence	Concentration
eNOS–F	5'-CCCAGGAGAGATCCACCTCA-3'	150 nM
eNOS–R	5'-CAGACACCGTAGTGCAGAGGG-3'	
nNOS–F	5'-CCGGTACGGGCATTGCTCCC-3	150 nM
nNOS–R	5'-CATGCGGCCTCCTTTGAG-3'	
PDE5 –F	5'-GGAAATGGTGGGACCTTCACT-3'	150 nM
PDE5 –R	5'-AAGAACAATACCACAGAATGCCA-3'	
TH–F	5'-CCCAGTTCTCCCAGGACATTG-3'	150 nM
TH–R	5'-CACAGCCCAAACTCCACAGTG-3'	
α_1A_-adr.–F	5'-ATGCCCATTGGGTCCTTCTT-3'	150 nM
α_1A_-adr.–R	5'-AGGGTTGATGCAACTATTTAGGTAC-3'	
α_1B_-adr.–F	5´- CTCCCACTTGGCTCCCTGTT-3´	150 nM
α_1B_-adr.–R	5´- CCAGCCAGAACACTACCTTGAAT-3	
α_1D_-adr.–F	5´-CCTGCCTCTGGGTTCTCTGTT-3´	150 nM
α_1D_-adr.–R	5´-GATGAGCGGGTTCACACAGC-3´	
β-actin–F	5′-ACTGCCGCATCCTCTTCCT-3′	70 nM
β-actin–R	5′-GAACCGCTCGTTGCCAATA-3′	
GAPDH–F	5′-TGCACCACCAACTGCTTA-3′	70 nM
GAPDH–R	5′-GGATGCAGGGATGATGTTC-3′	

F, foward; R, reverse; eNOS, endothelial nitric oxide synthase; nNOS, neuronal nitric oxide synthase; PDE5, phosphodiesterase type 5; TH, tyrosine hydroxylase; adr, adrenoceptor.

### Western blot analysis

Cavernosal tissues were homogenized in lysis buffer and centrifuged at 12,000 g for 20 minutes at 4°C. Total penile homogenates (75 μg total protein) were run on 4–20% Tris-HCl gels (Bio-Rad Laboratories, Hercules, CA, USA), transferred to a nitrocellulose membrane. Nonspecific binding sites were blocked with with 5% nonfat dry milk (Bio-Rad) in Tris-buffered saline/Tween for 1 hour at 24°C. Membranes were incubated overnight at 4°C with the following antibodies: polyclonal anti-tyrosine hydroxylase, anti-tyrosine hydroxylase phosphorylate at Ser^31^ (p-TH, 1:500, Cell Signaling Technology, Danvers, MA, USA) and monoclonal anti-β-actin (1:7000; Sigma-Aldrich, St. Louis, MO, USA). Densitometry was performed using the Image J Software (National Institute of Health, Bethesda-MD, USA). Quantified densitometry results of p-TH and total tyrosine hydroxylase were normalized to total tyrosine hydroxylase and β-actin, respectively.

### Drugs and chemicals

ACh, atropine, guanethidine, L-NAME, phenylephrine, prazosin and SNP were obtained from Sigma-Aldrich (St Louis, MO, USA), prepared in deionized water and stored in aliquots at -20°C. DHE (Sigma-Aldrich) stock solutions was prepared in dimethyl sulfoxide and stored in aliquots at -20°C. Dilutions were prepared immediately before use. All reagents used were of analytical grade.

### Statistical analysis

The GraphPad Prism Program (GraphPad Software Inc.) was used for statistical analysis. Data are expressed as the mean ± SEM of N experiments. Statistical comparisons were made using Student’s unpaired *t*-test. A value of P <0.05 was considered statistically significant.

## Results

### Contractile cavernosal responses induced by adrenergic nerve stimulation

**Fig 1:**
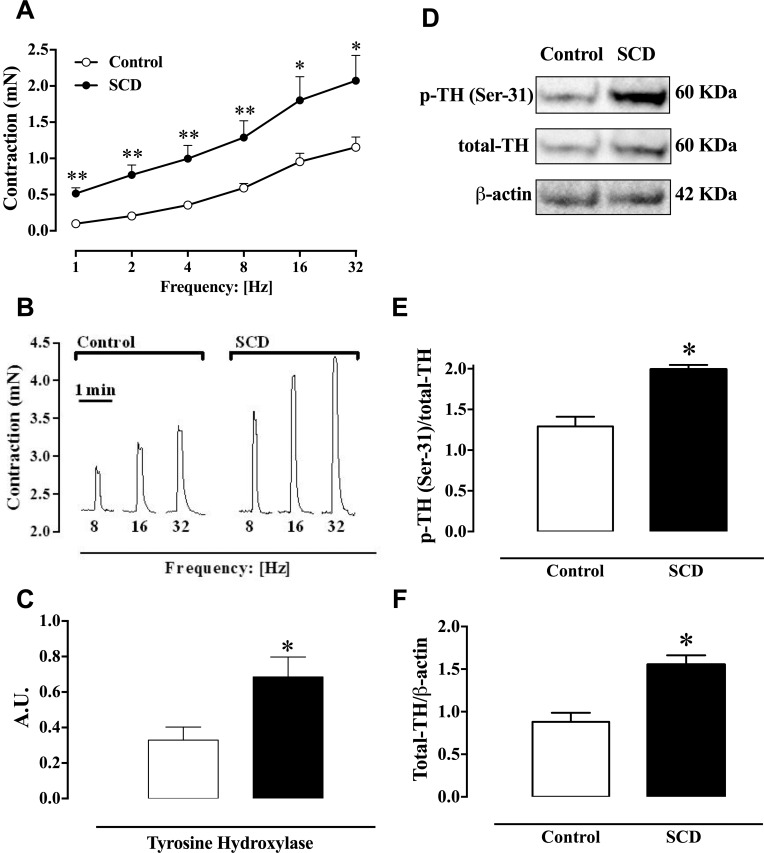
(**A**) Contractile responses to EFS (1–32 Hz) and (**B**) representative tracings of contractile response to EFS in corpus cavernosum strips from control and SCD mice. Data are shown in mN, and represent the mean ± SEM of 5 to 6 mice in each group. (**C**) mRNA expression of tyrosine hydroxylase in the homogenates of corpus cavernosum from control and SCD mice. (**D**) Representative blotting densitometric of tyrosine hydroxylase (TH) phosphorylated at Ser^31^ (p-TH), total TH and β-actin in the homogenates of corpus cavernosum from control and SCD mice detected by Western blotting. Protein values for (**E**) p-TH/total TH and (**F**) total TH/β-actin. Data represent the mean ± SEM of 4–5 mice in each group. Single asterisk indicates p <0.05 vs control group. Double asterisks indicate p <0.01 vs control group. (Student’s unpaired *t*-test).

To determine the contractile responses due to stimulation of adrenergic nerves, EFS experiments were conducted in the presence of N^ω^-nitro-L-arginine methyl ester (10^−4^ M, nonspecific NOS inhibitor) and atropine (10^−6^ M, nonselective muscarinic antagonist) to abolish the activation of nitrergic and cholinergic nerves, respectively. EFS (1–32 Hz) produced frequency-dependent corpus cavernosum contractions in both control and SCD mice (Fig 1A). In the cavernosal strips from SCD mice, EFS-induced contractions were significantly (P < 0.05) increased at all frequencies tested, when compared to the control group (Fig 1A). EFS-induced contractions were fully abolished by either the α-adrenoceptor antagonist prazosin (10^−6^ M; *n* = 5) or the sympathoinhibitory drug, guanethidine (3 × 10^−5^ M; *n* = 5), confirming that nerve-induced cavernosal contractile responses are mediated exclusively by noradrenaline release. Typical traces of contractile responses to EFS are shown in Fig 1B.

### mRNA expression for tyrosine hydroxylase, protein expression for total tyrosine hydroxylase and tyrosine hydroxylase phosphorylated at Ser-31 in corpus cavernosum

The mRNA expression for the tyrosine hydroxylase in cavernosal tissues was about 2-fold higher (P < 0.05) in SCD corpus cavernosum, compared with the control group (Fig 1C).

The protein expression for tyrosine hydroxylase phosphorylated at Ser-31 and total tyrosine hydroxylase was significantly higher (P < 0.05) by approximately 54% and 74% in cavernosal tissues from SCD group, in comparison with the control group, respectively (Fig 1E and 1F).

### Contractile cavernosal responses induced by phenylephrine and α_1_ adrenoceptor subtypes

**Fig 2:**
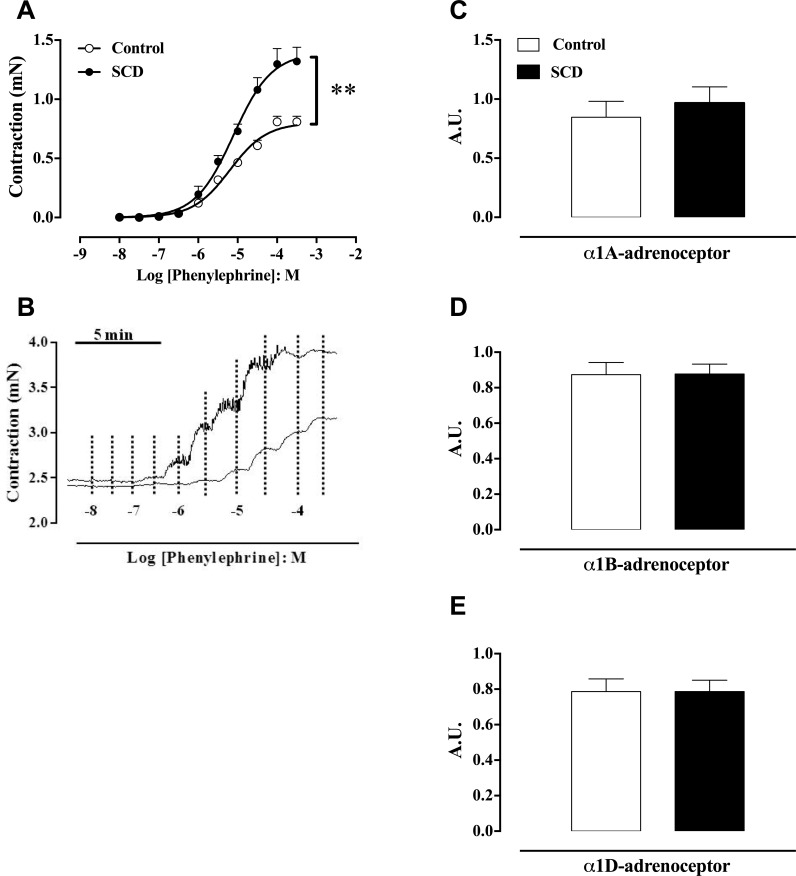
(**A**) Contractile responses to phenylephrine and (**B**) representative tracings of contractile response to phenylephrine in corpus cavernosum strips from control and SCD mice. Data are shown in mN, and represent the mean ± SEM of 5 to 6 mice in each group. mRNA expressions of (**C**) α_1A_- (**D**), α_1B_- (**E**), α_1D_-adrenoceptors in corpus cavernosum from control and SCD mice. The mRNA expression level of each gene was normalized by GAPDH and β-actin expression. Data represent the mean ± SEM of 5 mice in each group. Values are expressed in arbitrary units (A.U.). Single asterisk indicates p <0.05 vs control group. (Student’s unpaired *t*-test).

The α1-adrenoceptor agonist phenylephrine (10^−8^ to 3 x 10^−4^ M) induced concentration-dependent corpus cavernosum contractions in both control and SCD mice (Fig 2A). The maximal responses (E_max_) were significantly greater (P <0.01) in the corpus cavernosum of SCD, in comparison with control mice (Fig 2A, Table 2). No significant differences in pEC_50_ for phenylephrine were found between groups (Fig 2A, Table 2). Typical traces of contractile responses to phenylephrine are shown in Fig 2B, respectively.

**Table 2:** Potency (pEC_50_) and maximal response (E_max_) values obtained from concentration–response curves for phenylephrine (PE; 10^−8^ to 3 x 10^−4^ M), acetylcholine (ACh; 10^−9^ to 10^−5^ M), and sodium nitroprusside (SNP; 10^−8^ to 10^−5^ M) in cavernosal strips from control and SCD mice. Data represent the mean ± S.E.M. for 6 to 9 mice in each group.

	Control		SCD	
	pEC_50_	E_max_	pEC_50_	E_max_
PE	5.20 ± 0.05	0.8 ± 0.4 mN	5.1 ± 0.08	1.3 ± 0.1 mN*
ACh	6.83 ± 0.07	50 ± 4%	6.78 ± 0.07	78 ± 6%*
SNP	6.33 ± 0.05	77 ± 5%	6.46 ± 0.04	96 ± 3%*

*P < 0.01 compared to the control group.

The mRNA expression for the α_1A_-, α_1B_- and α_1D_-adrenoceptor remained unchanged among groups (Fig 2, 2C, 2D and 2E, respectively).

### Relaxant responses to ACh and SNP in cavernosal tissue

**Fig 3:**
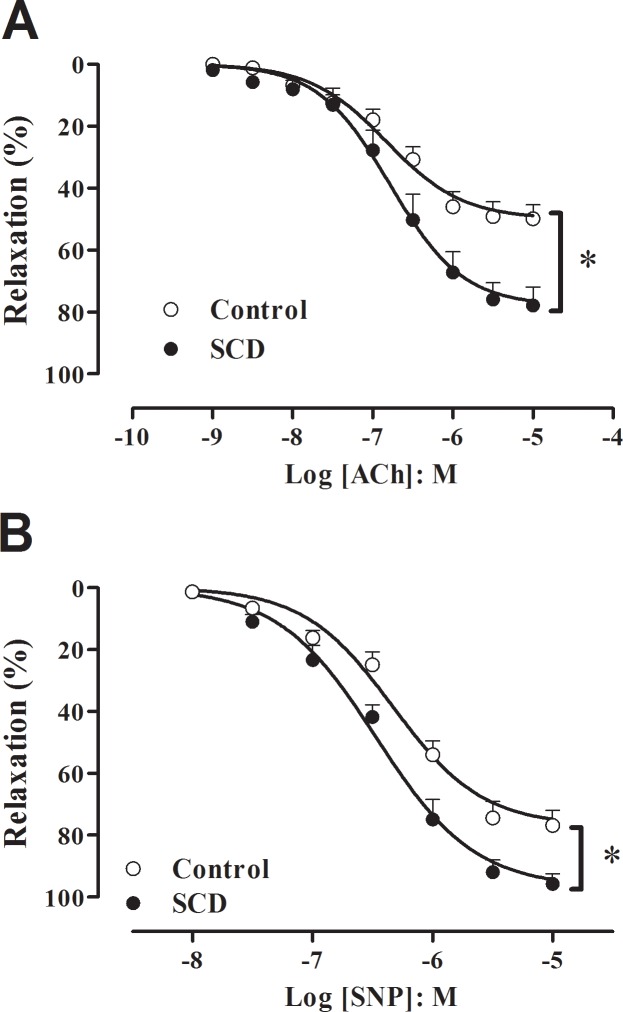
(**A**) Concentration-response relaxing curves to ACh, (**B**) SNP in corpus cavernosum strips from control and SCD mice. Data were calculated relative to the maximal changes from the contraction produced by phenylephrine (10^−5^ M for control mice and 3 × 10^−6^ M for SCD mice) in each tissue, which was taken as 100%. Data represent the mean ± SEM for 6 to 9 mice in each group. Single asterisk indicates p <0.01 vs control group. (Student’s unpaired *t*-test).

The addition of phenylephrine (10^−5^ M for control and 3x 10^−6^ M for SCD mice) to the tissue bath caused submaximal contractions of cavernosal segments that did not significantly differ between SCD (0.47 ± 0.03 mN) and control groups (0. 46 ± 0.03 mN) (N = 20–22). The cumulative addition of ACh (10^−9^ to 10^−5^ M) produced concentration-dependent relaxations in both groups (Fig 3A), but the maximal response (E_max_) was significantly higher in SCD, compared with control mice (P < 0.01; Fig 3A; Table 2). No significant differences in potency (pEC_50_) for ACh were found between groups (Table 2).

The cumulative addition of SNP (10^−8^ to 10^−5^ M) also produced concentration-dependent relaxations in corpus cavernosum from control and SCD mice, but again the E_max_ was significantly higher (P < 0.01) in SCD, compared to control mice (Fig 3B; Table 2). The pEC_50_ for SNP remained unchanged among groups (Table 2).

### Nitrergic relaxations in mouse corpus cavernosum

**Fig 4:**
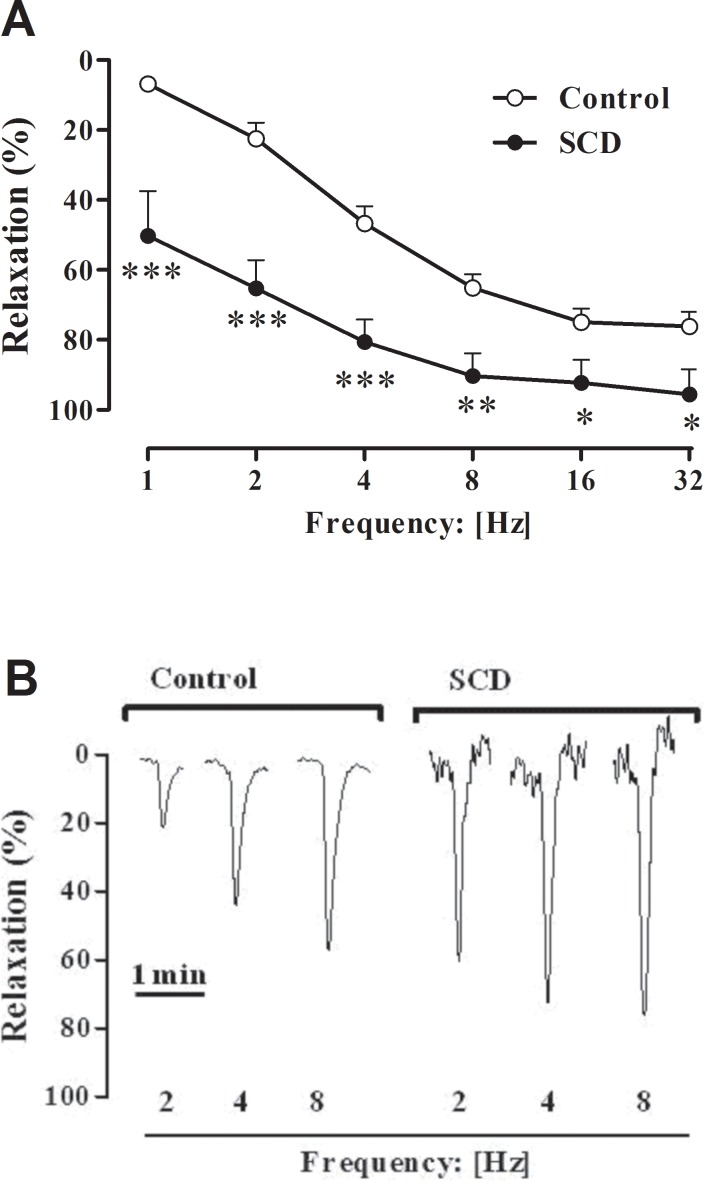
(**A**) Relaxation responses to EFS in corpus cavernosum strips from control and SCD mice. (**B**) Representative tracings of the relaxation response to EFS. Data were calculated relative to the maximal changes from the contraction produced by phenylephrine (10^−5^ M for control mice and 3 × 10^−6^ M for SCD mice) in each tissue, which was taken as 100%. Data represent the mean ± SEM of 5 to 8 mice in each group. Single asterisk indicates p <0.05 vs control group. Double asterisks indicate p <0.01 vs control group. Triple asterisks indicate p <0.001 vs control group. (Student’s unpaired *t*-test).

EFS (1 to 32 Hz) of cavernosal tissues pretreated with guanethidine (3 x 10^−5^ M) and atropine (10^−6^ M) caused frequency-dependent corpus cavernosum relaxations in both control and SCD mice (Fig 4A). A marked increase in the corpus cavernosum relaxations from SCD, in comparison with control mice, was observed at all frequencies studied (Fig 4A). Typical traces of relaxant responses to EFS are shown in Fig 4B

### mRNA expressions for eNOS, nNOS and PDE5

**Fig 5:**
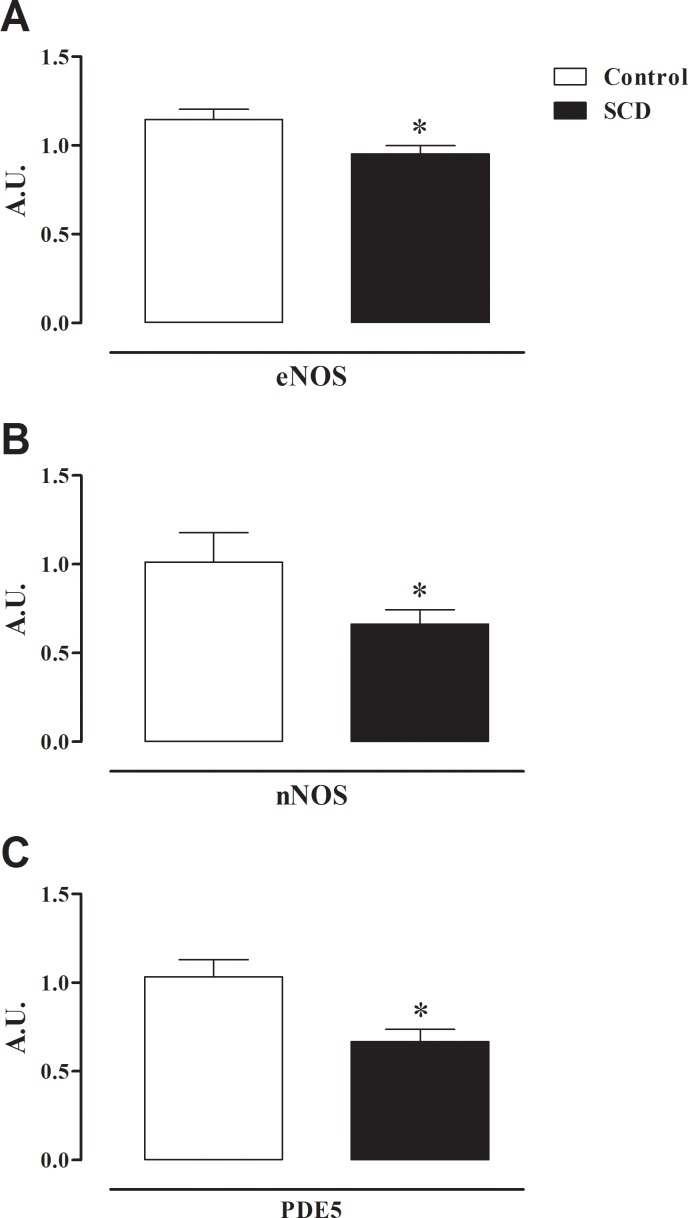
(**A**) mRNA expressions of eNOS, (**B**) nNOS and (**C**) PDE5 in corpus cavernosum from control and SCD mice. The mRNA expression level of each gene was normalized by GAPDH and β-actin expression. Data represent the mean ± SEM of 5 mice in each group. Values are expressed in arbitrary units (A.U.). Single asterisk indicates p <0.05 vs control group. (Student’s unpaired *t*-test).

The mRNA expressions for eNOS, nNOS and PDE5 were significantly reduced (P < 0.05) by approximately 17%, 35% and 36% in cavernosal tissues from SCD, in comparison with the control group, respectively (Fig 5).

### ROS levels in corpus cavernosum

**Fig 6:**
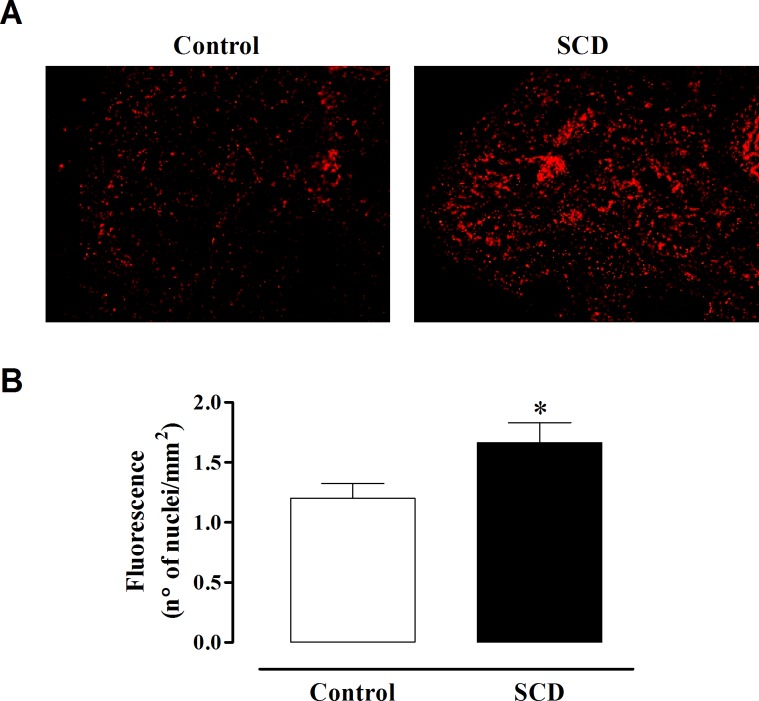
Reactive-oxygen species levels through hydroethidine-induced fluorescence in corpus cavernosum from control and SCD mice. (**A**) Representative and (**B**) quantitative analysis for hydroethidine-fluorescence photomicrographs of microscopic sections of corpus cavernosum. Data represent the mean ± SEM of 6 mice in each group. Single asterisk indicates p <0.05 vs control group. (Student’s unpaired *t*-test).

To evaluate the ROS levels in cavernosal tissue, we performed hydroethidine imaging of fresh frozen sections of corpus cavernosum preparations of control and SCD mice. The hydroethidine signal was 38% higher (P < 0.05) in the transversal cross-section of corpus cavernosum of SCD, compared to control mice (Fig 6).

## Discussion

In the present study, we show that Townes SCD mice display alterations in the contractile and relaxant mechanisms of the erectile tissue. The contractile responses mediated by sympathetic neurotransmission and α_1_-adrenoceptor activation were greater in corpus cavernosum from SCD mice, and the relaxant responses mediated by activation of the NO-cGMP signaling pathway were amplified in these animals.

Noradrenaline derived from the sympathetic nerves is responsible for maintaining the penis in the flaccid state via cavernosal smooth muscle contraction [8]. In mouse cavernosal strips, adrenergic nerve-mediated contractile responses are clearly seen under NO synthesis blockade and muscarinic receptor antagonism. Using N^ω^-nitro-L-arginine methyl ester and atropine to allow EFS-induced contractions caused by stimulation of adrenergic nerves only, we showed that the sympathetic cavernosal contractions were significantly greater in Townes SCD mice compared to control mice. Increases in immunostaining and mRNA expression for tyrosine hydroxylase in corpus cavernosum have been associated with sympathetic hyperactivity in diabetes and aging [14,20]. In our study, the mRNA and protein expression of tyrosine hydroxylase was increased in Townes SCD, compared with the control group, as well as tyrosine hydroxylase phosphorylation at its positive regulatory site Ser-31 [21]. Therefore, in this context, it reasonable suggest that the greater noradrenergic-induced corpus cavernosum contractions (EFS) in Townes SCD mice are due to an increased nerve-evoked noradrenaline release. Unfortunately, we have not measure noradrenaline release in penises of SCD and control mice.

Sympathetic neurotransmission is negatively modulated by endogenous NO [22,23]. Accordingly, NO synthesis inhibition enhances cavernosal contractions produced by sympathetic nerve stimulation, which reflect the removal of the relaxation normally caused by NO [14], associated with increased release of norepinephrine from sympathetic terminations [24,25]. In erectile tissue, decreased NO bioavailability leads to upregulation of tyrosine hydroxylase mRNA expression [14]. In our study, increased mRNA expression of tyrosine hydroxylase in the corpus cavernosum from Townes SCD mice may be the consequence of lower NO bioavailability. Moreover, corpus cavernosum from Townes SCD mice displayed increased contractions in response to direct α1-adrenoceptor activation with phenylephrine. It is likely that the lower NO availability in the cavernosal smooth muscle favors the contractile responses downstream of the α1-adrenoceptor. This is consistent with our findings showing that α_1A_-, α_1B_-, α_1D_-adrenoceptor mRNA expressions remain unchanged between groups, thus excluding a role for upregulation of these receptors in mediating the sympathetic hyperactivity in SCD mice. Consistent with our findings, a previous study showed an increased contractile response to α1-adrenergic receptor activation in the corpus cavernosum of eNOS and nNOS gene-deficient mice [26].

The NO-cGMP signaling pathway is the most important pathway for mediating erectile function [11]. The intracellular cGMP levels in cavernosal smooth muscle is dependent on the balance between its production by soluble guanylate cyclase and its breakdown by PDE5 [8]. Selective PDE5 inhibition results in the accumulation of intracellular cGMP and increases NO-dependent penile tissue relaxations [12]. Cyclic GMP, in turn, is a positive regulator of PDE5 gene expression in cavernosal smooth muscle [27]. The enhanced cavernosal relaxations to NO released from both endothelial cells (ACh) and nitrergic nerves (EFS) in Townes SCD mice were accompanied by lower mRNA expression for PDE5 in the cavernosal tissue. Interestingly, significantly lower mRNA expressions for eNOS and nNOS in the corpus cavernosum of Townes SCD mice was observed, which is consistent with a previous study showing that corpus cavernosum from eNOS- or both eNOS- and nNOS gene-deficient mice displays downregulation of PDE5 protein expression and activity [28]. Therefore, the reduced metabolism of cGMP by PDE5 in Townes SCD mice may lead to the enhancement of NO-dependent cavernosal relaxations. Unfortunately, we have not measure cGMP levels in corpus cavernosum of Townes SCD. The inorganic compound SNP is an agent that releases NO in biological systems by nonenzymatic and enzymatic mechanisms [29]. In our study, SNP-induced cavernosal relaxations were significantly increased in the Townes SCD group. This is consistent with our findings showing that mRNA expressions for PDE5 is lower in the erectile tissue from Townes SCD. Decreased mRNA/protein expression of PDE5 in the erectile tissue have been reported in the Berkeley SCD model [28,30] and SCD patients with a history of priapism [31], as well as higher relaxing responses to endothelium-dependent and endothelium-independent agents [32]. Oxidative stress, an imbalance between the production and elimination of ROS, is a major factor in the pathogenesis of SCD, and has been reported to play an important role in the pathophysiology of hemolysis, vaso-occlusion and ensuing organ damage in this disease [16]. Elevated ROS production by about of 40% in the erectile tissues from Townes SCD was detected. Therefore, besides reduced NOS-derived NO, an excess of superoxide anion may react with NO, decreasing its bioavailability, and thus leading to PDE5 downregulation in corpus cavernosum. A limitation of our study is that we did not measure PDE5 protein expression in corpus cavernosum of Townes SCD, due to a limited amount of tissue.

## Conclusions

In summary, our study shows that Townes SCD mice display sympathetic hyperactivity, accompanied by upregulation of tyrosine hydroxylase expression, as well as enhanced NO-mediated cavernosal relaxations, associated with downregulation of eNOS, nNOS and PDE5. The elevated levels of ROS in corpus cavernosum from Townes SCD mice may act to upregulate the sympathetic machinery at the same time as reducing NO-cGMP pathway signaling.

## References

[1] Rees DC, Williams TN, Gladwin MT. Sickle-cell disease. The Lancet. 2010;376: 2018–2031. 10.1016/S0140-6736(10)61029-X 21131035

[2] Pászty C, Brion CM, Manci E, Witkowska HE, Stevens ME, Mohandas N, et al Transgenic knockout mice with exclusively human sickle hemoglobin and sickle cell disease. Science. 1997;278: 876–878. 9346488 10.1126/science.278.5339.876

[3] Wu L-C, Sun C-W, Ryan TM, Pawlik KM, Ren J, Townes TM. Correction of sickle cell disease by homologous recombination in embryonic stem cells. Blood. 2006;108: 1183–1188. 10.1182/blood-2006-02-004812 16638928 PMC1895869

[4] Kheirandish P, Chinegwundoh F, Kulkarni S. Treating stuttering priapism. BJU Int. 2011;108: 1068–1072. 10.1111/j.1464-410X.2011.10367.x 21914108

[5] Salonia A, Eardley I, Giuliano F, Hatzichristou D, Moncada I, Vardi Y, et al European Association of Urology guidelines on priapism. Eur Urol. 2014;65: 480–489. 10.1016/j.eururo.2013.11.008 24314827

[6] Tamura M, Kagawa S, Kimura K, Kawanishi Y, Tsuruo Y, Ishimura K. Coexistence of nitric oxide synthase, tyrosine hydroxylase and vasoactive intestinal polypeptide in human penile tissue—a triple histochemical and immunohistochemical study. J Urol. 1995;153: 530–534. 10.1097/00005392-199502000-00077 7529339

[7] Hedlund P, Ny L, Alm P, Andersson KE. Cholinergic nerves in human corpus cavernosum and spongiosum contain nitric oxide synthase and heme oxygenase. J Urol. 2000;164: 868–875. 10953170 10.1097/00005392-200009010-00064

[8] Andersson K-E. Mechanisms of Penile Erection and Basis for Pharmacological Treatment of Erectile Dysfunction. Pharmacol Rev. 2011;63: 811–859. 10.1124/pr.111.004515 21880989

[9] Nakashima A, Hayashi N, Kaneko YS, Mori K, Sabban EL, Nagatsu T, et al Role of N-terminus of tyrosine hydroxylase in the biosynthesis of catecholamines. J Neural Transm Vienna Austria 1996. 2009;116: 1355–1362. 10.1007/s00702-009-0227-8 19396395

[10] Lue TF. Erectile dysfunction. N Engl J Med. 2000;342: 1802–1813. 10.1056/NEJM200006153422407 10853004

[11] Burnett AL, Musicki B. The nitric oxide signaling pathway in the penis. Curr Pharm Des. 2005;11: 3987–3994. 16378505 10.2174/138161205774913381

[12] Carson CC, Lue TF. Phosphodiesterase type 5 inhibitors for erectile dysfunction. BJU Int. 2005;96: 257–280. 10.1111/j.1464-410X.2005.05614.x 16042713

[13] Azadzoi KM, Golabek T, Radisavljevic ZM, Yalla SV, Siroky MB. Oxidative stress and neurodegeneration in penile ischaemia. BJU Int. 2010;105: 404–410. 10.1111/j.1464-410X.2009.08717.x 19549113

[14] Silva FH, Lanaro C, Leiria LO, Rodrigues RL, Davel AP, Claudino MA, et al Oxidative stress associated with middle aging leads to sympathetic hyperactivity and downregulation of soluble guanylyl cyclase in corpus cavernosum. Am J Physiol—Heart Circ Physiol. 2014;307: H1393–H1400. 10.1152/ajpheart.00708.2013 25217652

[15] Muniz JJ, Leite LN, De Martinis BS, Carneiro FS, Tirapelli CR. Chronic ethanol consumption induces erectile dysfunction: Role of oxidative stress. Life Sci. 2015;141: 44–53. 10.1016/j.lfs.2015.09.017 26407475

[16] Nur E, Biemond BJ, Otten H-M, Brandjes DP, Schnog J-JB, the CURAMA Study Group. Oxidative stress in sickle cell disease; pathophysiology and potential implications for disease management. Am J Hematol. 2011;86: 484–489. 10.1002/ajh.22012 21544855

[17] Silva FH, Leiria LO, Alexandre EC, Davel APC, Mónica FZ, De Nucci G, et al Prolonged therapy with the soluble guanylyl cyclase activator BAY 60–2770 restores the erectile function in obese mice. J Sex Med. 2014;11: 2661–2670. 10.1111/jsm.12682 25196910

[18] Toque HA, da Silva FH, Calixto MC, Lintomen L, Schenka AA, Saad MJ, et al High-fat diet associated with obesity induces impairment of mouse corpus cavernosum responses. BJU Int. 2011;107: 1628–1634. 10.1111/j.1464-410X.2010.09704.x 20942830

[19] Vandesompele J, De Preter K, Pattyn F, Poppe B, Van Roy N, De Paepe A, et al Accurate normalization of real-time quantitative RT-PCR data by geometric averaging of multiple internal control genes. Genome Biol. 2002;3: RESEARCH0034 12184808 10.1186/gb-2002-3-7-research0034 PMC126239

[20] Morrison JFB, Pallot DJ, Sheen R, Dhanasekaran S, Mensah-Brown EPK. The effects of age and streptozotocin diabetes on the sympathetic innervation in the rat penis. Mol Cell Biochem. 2007;295: 53–58. 10.1007/s11010-006-9271-y 16944308

[21] Moy LY, Tsai L-H. Cyclin-dependent kinase 5 phosphorylates serine 31 of tyrosine hydroxylase and regulates its stability. J Biol Chem. 2004;279: 54487–54493. 10.1074/jbc.M406636200 15471880

[22] Schwarz P, Diem R, Dun NJ, Förstermann U. Endogenous and exogenous nitric oxide inhibits norepinephrine release from rat heart sympathetic nerves. Circ Res. 1995;77: 841–848. 7554131 10.1161/01.res.77.4.841

[23] Cellek S, Moncada S. Nitrergic control of peripheral sympathetic responses in the human corpus cavernosum: a comparison with other species. Proc Natl Acad Sci U S A. 1997;94: 8226–8231. 9223343 10.1073/pnas.94.15.8226 PMC21585

[24] Kolo LL, Westfall TC, Macarthur H. Nitric oxide decreases the biological activity of norepinephrine resulting in altered vascular tone in the rat mesenteric arterial bed. Am J Physiol Heart Circ Physiol. 2004;286: H296–303. 10.1152/ajpheart.00668.2003 14684362

[25] Macarthur H, Wilken GH, Westfall TC, Kolo LL. Neuronal and non-neuronal modulation of sympathetic neurovascular transmission. Acta Physiol Oxf Engl. 2011;203: 37–45. 10.1111/j.1748-1716.2010.02242.x PMC3139802 21362154

[26] Nangle MR, Cotter MA, Cameron NE. An in vitro investigation of aorta and corpus cavernosum from eNOS and nNOS gene-deficient mice. Pflüg Arch Eur J Physiol. 2004;448: 139–145. 10.1007/s00424-003-1232-7 14722775

[27] Lin C-S, Chow S, Lau A, Tu R, Lue TF. Human PDE5A gene encodes three PDE5 isoforms from two alternate promoters. Int J Impot Res. 2002;14: 15–24. 10.1038/sj.ijir.3900802 11896473

[28] Champion HC, Bivalacqua TJ, Takimoto E, Kass DA, Burnett AL. Phosphodiesterase-5A dysregulation in penile erectile tissue is a mechanism of priapism. Proc Natl Acad Sci U S A. 2005;102: 1661–1666. 10.1073/pnas.0407183102 15668387 PMC547836

[29] Bonaventura D, Lunardi CN, Rodrigues GJ, Neto MA, Bendhack LM. A novel mechanism of vascular relaxation induced by sodium nitroprusside in the isolated rat aorta. Nitric Oxide Biol Chem Off J Nitric Oxide Soc. 2008;18: 287–295. 10.1016/j.niox.2008.02.004 18307997

[30] Ning C, Wen J, Zhang Y, Dai Y, Wang W, Zhang W, et al Excess adenosine A2B receptor signaling contributes to priapism through HIF-1α mediated reduction of PDE5 gene expression. FASEB J Off Publ Fed Am Soc Exp Biol. 2014;28: 2725–2735. 10.1096/fj.13-247833 PMC4021439 24614760

[31] Lagoda G, Sezen SF, Cabrini MR, Musicki B, Burnett AL. Molecular analysis of erection regulatory factors in sickle cell disease associated priapism in the human penis. J Urol. 2013;189: 762–768. 10.1016/j.juro.2012.08.198 22982429 PMC4478587

[32] Claudino MA, Franco-Penteado CF, Corat MAF, Gimenes AP, Passos LAC, Antunes E, et al Increased cavernosal relaxations in sickle cell mice priapism are associated with alterations in the NO-cGMP signaling pathway. J Sex Med. 2009;6: 2187–2196. 10.1111/j.1743-6109.2009.01337.x 19493282

